# Capacity and establishment rules govern the number of nonnative species in communities of ground‐dwelling invertebrates

**DOI:** 10.1002/ece3.10856

**Published:** 2024-03-13

**Authors:** Michael Kaspari, Michael D. Weiser, Cameron D. Siler, Katie E. Marshall, Sierra N. Smith, Katherine M. Stroh, Kirsten M. de Beurs

**Affiliations:** ^1^ Geographical Ecology Group, Department of Biology University of Oklahoma Norman Oklahoma USA; ^2^ Conservation Ecology Center Smithsonian's National Zoo and Conservation Biology Institute Front Royal Virginia USA; ^3^ Sam Noble Oklahoma Museum of Natural History University of Oklahoma Norman Oklahoma USA; ^4^ Department of Zoology University of British Columbia Vancouver British Columbia Canada; ^5^ Laboratory of Geo‐Information Science and Remote Sensing Wageningen University and Research Wageningen The Netherlands

**Keywords:** capacity rules, establishment rules, geographical ecology, habitat, invertebrate communities, net primary productivity, pitfall traps, species invasions, temperature, vehicular traffic

## Abstract

Nonnative species are a key agent of global change. However, nonnative invertebrates remain understudied at the community scales where they are most likely to drive local extirpations. We use the North American NEON pitfall trapping network to document the number of nonnative species from 51 invertebrate communities, testing four classes of drivers. We sequenced samples using the eDNA from the sample's storage ethanol. We used AICc informed regression to evaluate how native species richness, productivity, habitat, temperature, and human population density and vehicular traffic account for continent‐wide variation in the number of nonnative species in a local community. The percentage of nonnatives varied 3‐fold among habitat types and over 10‐fold (0%–14%) overall. We found evidence for two types of constraints on nonnative diversity. Consistent with Capacity rules (i.e., how the number of niches and individuals reflect the number of species an ecosystem can support) nonnatives increased with existing native species richness and ecosystem productivity. Consistent with Establishment Rules (i.e., how the dispersal rate of nonnative propagules and the number of open sites limits nonnative species richness) nonnatives increased with automobile traffic—a measure of human‐generated propagule pressure—and were twice as common in pastures than native grasslands. After accounting for drivers associated with a community's ability to support native species (native species richness and productivity), nonnatives are more common in communities that are regularly seasonally disturbed (pastures and, potentially deciduous forests) and those experiencing more vehicular traffic. These baseline values across the US North America will allow NEON's monitoring mission to document how anthropogenic change—from disturbance to propagule transport, from temperature to trends in local extinction—further shape biotic homogenization.

## Graphical abstract

We use the North American NEON pitfall trapping network to document the number of nonnative species from 51 invertebrate communities, testing four classes of drivers. We found evidence for two types of constraints on nonnative diversity. Consistent with Capacity rules (i.e., how the number of niches and individuals reflect the number of species an ecosystem can support) nonnatives increased with existing native species richness and ecosystem productivity. Consistent with Establishment Rules (i.e., how the dispersal rate of nonnative propagules and the number of open sites limits nonnative species richness) nonnatives increased with automobile traffic—a measure of human‐generated propagule pressure—and were twice as common in pastures than native grasslands.

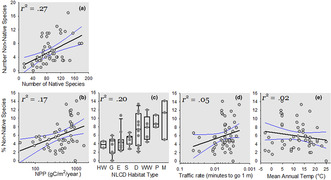

## 1 INTRODUCTION

**FIGURE 1:**
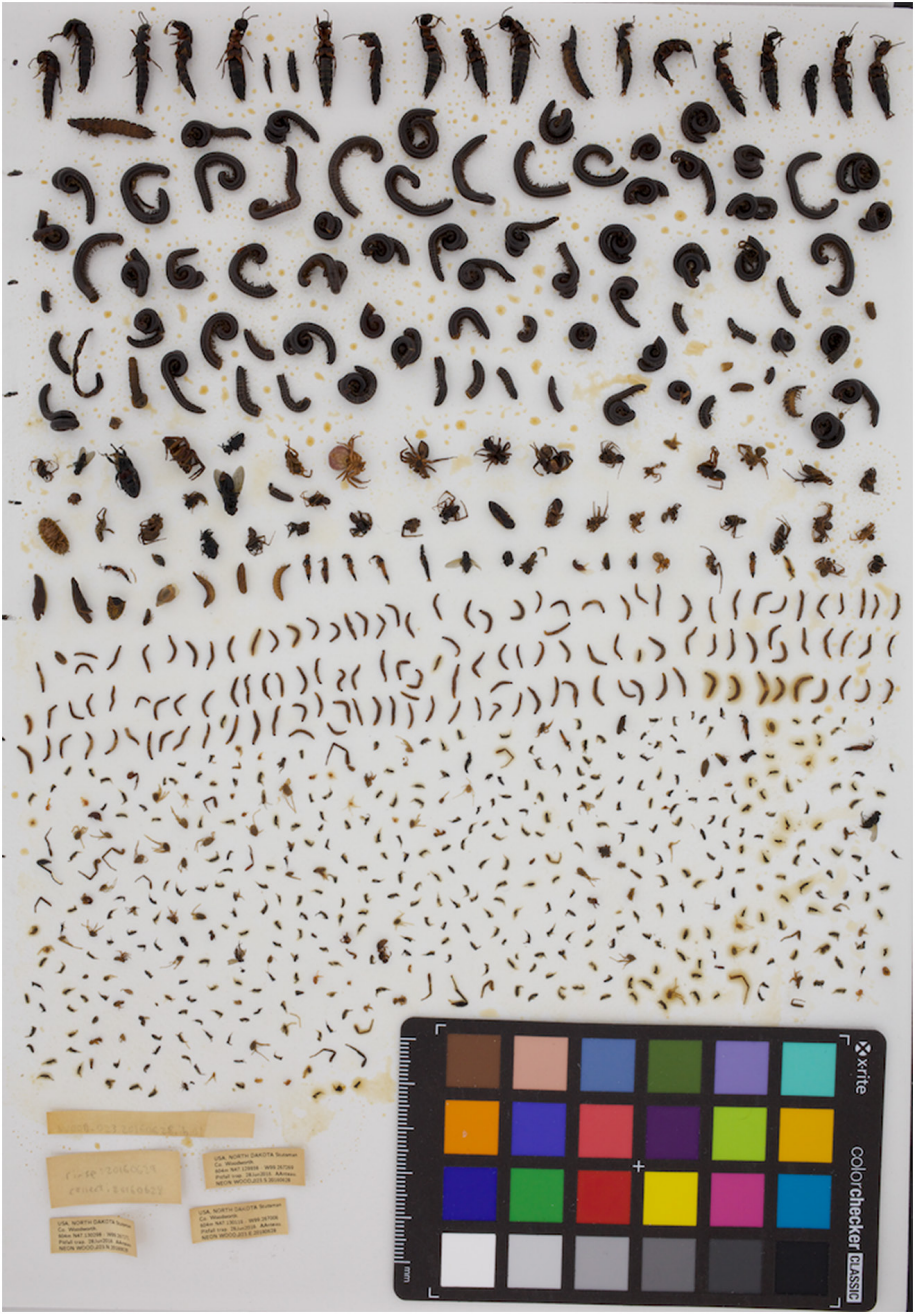
A single pitfall sample from the NEON network, with each individual separated by hand to create white space for image analysis.

In an era of insect declines the expanding ranges of nonnative species frequently generate richer, more uniform biota (an era deemed the Homogocene, Rahel, 2000; Rosenzweig, 2001). At the continental scale, invertebrate invasions are projected to strongly increase through 2050 (Seebens et al., 2021). Moreover, given the numerous impacts of nonnative species on diversity and ecosystem function (Bohlen et al., 2004; Jochum et al., 2021; Simberloff, 2013) there is a growing need for systematic data on Earth's changing ecological communities and the drivers of this change (Wagner et al., 2021). NEON (National Ecological Observatory Network)—the 30‐year network monitoring US ecosystems (Levan, 2020)—consists of one continental network that would realistically test such global drivers of change. NEON samples ground invertebrates from Alaska to Puerto Rico across all major US terrestrial habitat types (Figure 1). By sampling at community (e.g., <1 km^2^) scales—spatial grains where local extirpations are most likely to occur (e.g., Levine, 2000)—NEON builds on foundational studies of nonnative distribution that use species lists from island or geographic meridians (Hawkins & Porter, 2003; Sax & Gaines, 2008).

A variety of mechanisms can shape the richness, traits, and impact of nonnative species—a recent review (Enders et al., 2020) tallied 39 such hypotheses. In contrast to Enders more universalist approach, our schema focus on processes that regulate the number of nonnative species that can and do establish in a community. Like Brown's (1981) Capacity Rule, Ender and colleague's “Resource Availability Cluster” holds a foundational place in in invasion biology; likewise, our Establishment Rule approximates their “Propagule Cluster” describing the rate of propagule arrival. Much of Ender and colleague's other three clusters describe the features of populations (“Trait Cluster,” “Darwin's Cluster”), and their population interactions (“Biotic Interaction Cluster”) that determine the composition of invaded communities. Put another way, Capacity and Establishment rules, like island biogeography, focus on a neutral assembly rules (e.g., Hubbell, 2001) that ignore species differences, and predict species numbers, while Enders other three clusters are more firmly grounded in Niche Theory (e.g., Chase, 2011) and predict species composition.

**FIGURE 2:**
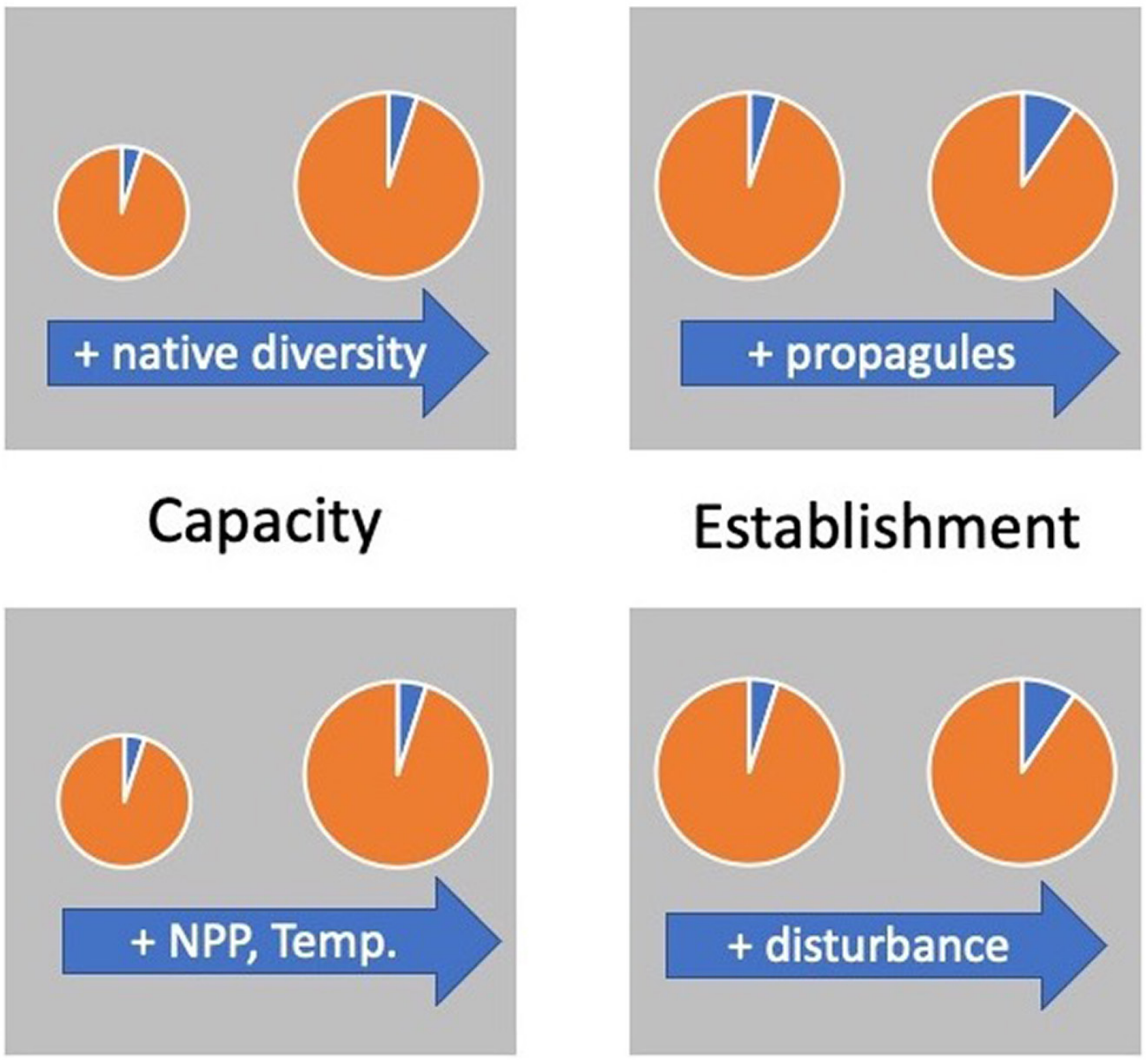
Hypotheses for the number of nonnative species supported by a community. The size of the circle represents the total number of species, broken down into native (orange) and nonnative (blue). Mechanisms can be grouped into Capacity rules (left)—those that regulate the number of species regardless of their native status—and Establishment rules (right) those that favor the successful invasion of nonnatives.

Here we take a simplified approach similar to the Theory of Island Biogeography (MacArthur & Wilson, 1967) that focuses on the question “What regulates the number of nonnative species in an invertebrate community” positing that the answer lies in the size/capacity of the island, and the rate that propagules can arrive and establish (Figure 2). The first—Brown (1981)'s Capacity Rules—document processes that constrain the number of extant individuals and species of any community—natives and nonnatives alike (akin to the axioms “the rich get richer” and “a rising tide lifts all boats”). If nonnative and native richness are constrained by the same drivers the two should positively covary (Elton, 1958; Levine, 2000; Sax & Gaines, 2008). Under this logic, the question of what regulates the number of nonnatives must effectively account first for the number of native species (e.g., through residuals).

One specific Capacity Rule is driven by the constraint on community abundance by the availability of energy (Brown, 1981; Hurlbert & Stegen, 2014; Srivastava & Lawton, 1998; Wright, 1983). Net Primary Productivity (NPP, gC/m^2^/year) is one such ecosystem measure of energy—effectively glucose accessible to consumers—that can limit community abundance. If a community's individuals are close to equilibrium with NPP, then NPP should predict abundance as it does in communities of ants (Kaspari, O'Donnell, & Kercher, 2000) and ungulates (McNaughton et al., 1989). Moreover, as the energy needed to construct and maintain individuals *decreases*, so should abundance, increasing the probability of local species extirpation (Hurlbert, 2006; Hurlbert & Stegen, 2014; Srivastava & Lawton, 1998). A second form of energy—measured by temperature—can have similar downstream effects on abundance and diversity: for ectotherms, warmer temperatures can enhance abundance (Kaspari, Alonso, & O'Donnell, 2000) and, potentially, diversity. However, the unimodal shape of thermal performance curves (Huey & Kingsolver, 2019; Kingsolver & Huey, 2008) suggests the temperature–abundance relationship may flip, in turning flipping its effect on community diversity.

For a given community capacity, new, nonnative species must arrive via propagules. Establishment Rules (Gaines & Roughgarden, 1985; Suarez et al., 2005; Tilman, 1997) focus on how the arrival rate of nonnative propagules—and the availability of open sites when they do arrive—combine to increase the fraction of nonnatives in a local community (Figure 2). Establishment constraints go beyond those drivers that enhance native and nonnative diversity alike to focus on drivers that favor the dispersal and establishment of nonnatives. At least two features of the human landscape—human population density and vehicular traffic—have the potential to constrain the number of nonnative propagules delivered to a habitat (Dawson et al., 2017; Suarez et al., 2005; Von der Lippe & Kowarik, 2007). Once arrived, rates of disturbance that open up competitor free‐space can also enhance establishment (Levine, 2000; Simberloff, 2013).

The application of Environmental DNA (eDNA) approaches (i.e. quantitative PCR [qPCR], digital droplet PCR [ddPCR], metagenomic approaches, etc.) have become increasingly common in experimental and field‐based conservation and wildlife monitoring research programs, including quantification of terrestrial faunas (Mena et al., 2021). A key advantage of eDNA for quantifying collections of preserved samples is its non‐destructive nature. Here we quantify the nonnative and native composition of ground invertebrates from NEON's pitfall sample arrays. We extract, sequence, and identify to species taxa from the DNA in the alcohol of stored samples to generate a standardized geography of North American ground invertebrate communities.

We then use these 51 communities across continental US North America to evaluate hypothesized drivers of nonnative richness. Specifically, we focus first on Capacity Rules prediction that native richness covaries with nonnative richness and is further increased in ectotherm communities by increasing the food supply via primary productivity and its accessibility via temperature. We next test Establishment Rules that predict higher arrival rates of nonnative species in ecological communities embedded in landscapes with higher human population and vehicular traffic, and in more/less disturbed habitats (pastures and hay fields vs. natural grasslands).

## 2 MATERIALS AND METHODS

The National Ecological Observatory Network was constructed over a 5‐year period (2014–2019) for a proposed 30 years of monitoring abiotic and biotic changes across the ecosystems of the United States (Alaska, Hawaii, Puerto Rico, and the 48 contiguous states). At each site, NEON identifies up to four habitat types based on NLCD land cover codes (Fry et al., 2008) eight of which are represented here (Deciduous Forest, Evergreen Forest, Mixed Forest, Shrub/Scrub, Grassland/Herbaceous Sedge/Herbaceous, Pasture/Hay, Woody Wetlands). Ten pitfall trap arrays are distributed across the habitats and sampled across the growing season, defined as the weeks when average minimum temperatures exceed 4°C for 10 days and ending when temperatures remain below 4°C for the same period.

Each of the four traps in a pitfall array was a 473 mL plastic deli container ca. 11 cm in diameter and 7 cm deep containing 150–250 mL of 1:1 deionized water and propylene glycol (summarized in Levan, 2020). Traps are placed flush in the soil with a square cover 1.5 cm above the trap. Every 14 days, traps are emptied and replaced; the contents of all traps from an array are pooled and stored in 95% EtOH‐filled 50‐mL tubes.

**FIGURE 3:**
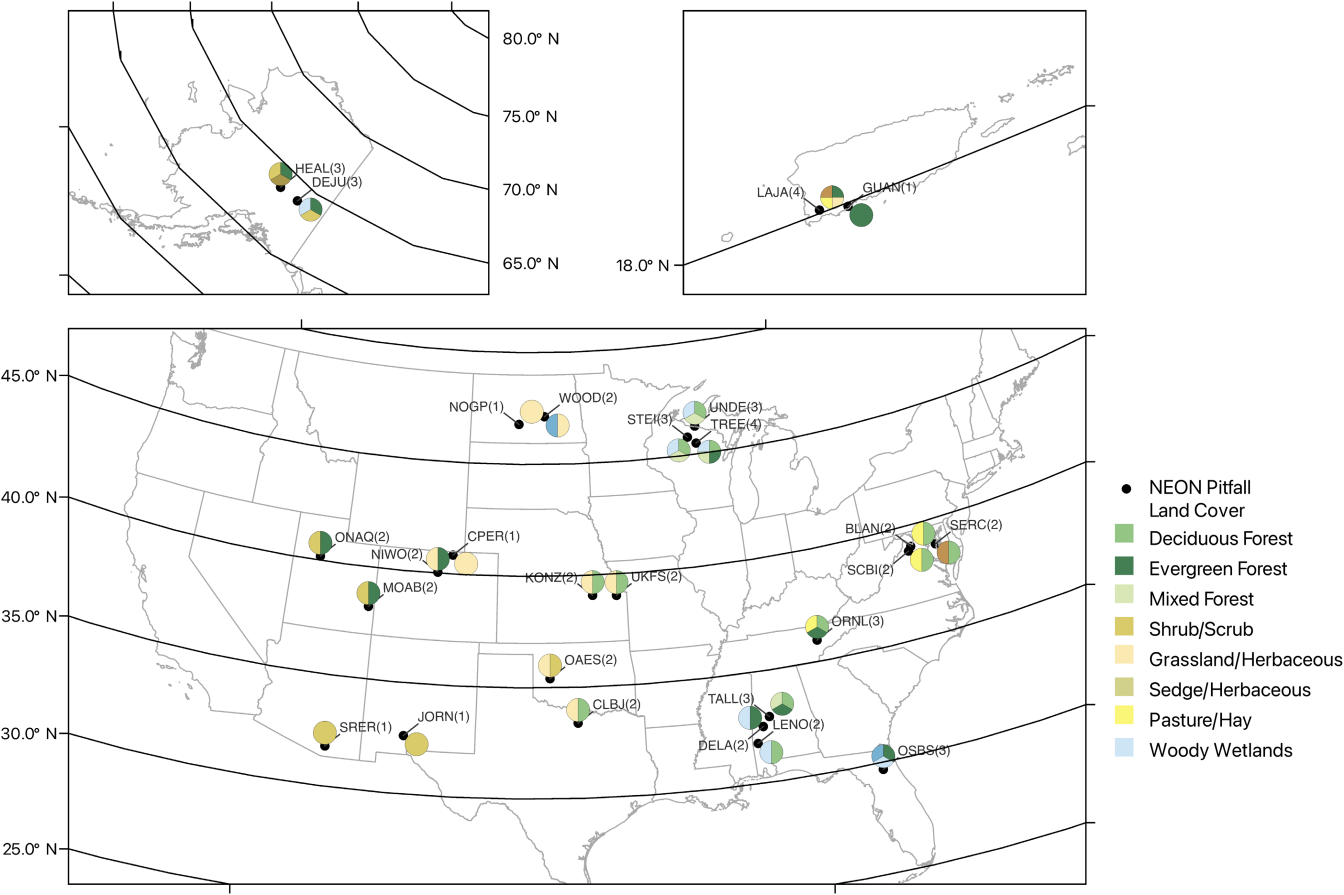
Map of our NEON sampling sites accompanied by a pie chart reflecting the number of NLCD habitat classes it supports. Altogether, we analyzed the richness of 51 site‐habitat based invertebrate communities.

For this analysis, we used samples (NEON Biorepository, 2023) from the 27 NEON sites that were active in the 2016 field season (Figure 3). We used pitfall arrays from the 8 NLCD land cover categories at each site for a total of 51 site‐habitat combinations. For the majority (44), each represents three pitfall samples pooled from samples taken early, in the middle, and at the end of the site's sampling season. Due to logistical difficulties at NEON, the remaining seven site‐habitat combinations come from two seasonal samples.

### 2.1 Molecular methods used to identify OTUs from storage ethanol

To identify taxa in the pitfall traps we used methods modified from current environmental DNA techniques (Taberlet et al., 2018) which allowed us to nondestructively sample DNA from the invertebrates using the ethanol in which they were preserved. All of the storage ethanol was vacuum filtered using a 50 mL Autofil® Vacuum Filtration System (Stellar Scientific) with a 0.45‐μm pore size. After filtering, we used sterilized scissors to cut the filter from the tube which was then stored at −20°C (see Appendix S1 for complete methods for DNA extraction, metagenomic barcoding, and taxonomic assignment).

The sequencing effort returned 2.7 million reads of which 1.4 million were assigned a species‐level identification with ≥97% similarity to reference sequences. We detected 6330 taxon‐by‐array occurrences from three focal phyla: Annelida, Arthropoda, and Mollusca. We removed occurrences with incomplete taxonomic assignment, leaving 3645 species‐by‐array occurrences from 1914 species.

### 2.2 Assigning nonnative status

We queried each species name × site occurrence against online databases including Bison (BISON, 2022), GBIF (Flemons et al., 2007), iNaturalist (Van Horn et al., 2018), BugGuide (Bartlett, 2003), and the World Spider Catalog (Platnick, 2010). Next Google Scholar searches (scholar.google.com) began with “Genus + species + U.S. state of interest”. Lacking any hits, we proceeded to “Genus + species + United States”, then “Genus + Species + distribution”, then “Genus + species”. The process was then repeated using Google (google.com). Criteria denoting nonnative status varied among sources (e.g., “invasive”, “naturalized”, etc.) but only when a species occurrence at one of our pitfall array sites was identified as such, we assigned it nonnative status.

We found 1340 confirmed native and 315 confirmed nonnative species across all 51 trap arrays sampled.

### 2.3 Quantifying driver variables

For each biweekly sample period we calculated the mean air temperature as the average of the daily minimum and maximum temperature over the 14 days preceding the date the trap sample was collected. We used the Daymet V4 database (Thorton et al., 2020) which provides gridded estimates of daily weather for most of North America. Daymet V4 interpolates and extrapolates daily meteorological station data from NOAA's Global Historical Climatology Network (GHCN). The spatial resolution of the gridded dataset is 1 × 1 km. This dataset was published as version 4 on April 8, 2021.

The MOD17A3HGF Version 6 product provides information about annual Net Primary Production (NPP) at 500‐meter (m) pixel resolution. Annual NPP is derived from the sum of all 8‐day Net Photosynthesis (PSN) products (MOD17A2H) from the given year. The PSN value is the difference of the Gross Primary Productivity (GPP) and the Maintenance Respiration (MR).

For human population density we used the Gridded Population of World Version 4 (GPWv4), Revision 11 (CIESIN, 2018). It models the distribution of global human population for the years 2000, 2005, 2010, 2015, and 2020 on 30 arc‐second (approximately 1 km) grid cells. Population is distributed to cells using proportional allocation of population from census and administrative units. We estimate the population density in a circle with a 100 km radius centered around each trap array.

For estimates of vehicular traffic flow, we used a global friction surface with a nominal year of 2019 (Weiss et al., 2020) to calculate the average global travel time in minutes per meter. The original dataset has a spatial resolution of 927.67 m. We estimate the rate of traffic flow in a circle with a 100 km radius centered around each trap array.

A note on the spatial nature of the NEON dataset and its subsequent analysis. It would be useful, given that the data are clearly spatially resolved, to cluster sites by their regional source pools and analyze this effect. However, this information is not reliably available from the data sources we use for our analysis and has therefore not been considered. Regarding spatial structure in our other drivers, NEON was built to represent *proportionately and uniformly* the distribution of North American ecosystem types (see map on Figure 3); this should minimize over and under‐sampling of particular ranges of driver variables, as might happen in a meta‐analysis of the available literature. Also, individual NEON sites are broken into nlcdClass habitat types, which in turn are determined by local topography, soil structure, and hydrology at each site. This habitat variety breaks down regional autocorrelation by generating an array of NPPs, temperatures, and diversity/composition among pitfall traps in a given site, any of which might include side by side deciduous forests, wetlands, and grasslands.

### 2.4 Statistics

We used SAS 9.4 program GLMSelect (SAS_Institute, 2013) to analyze how the above drivers account for the number of nonnative species across 51 communities. GLMSelect uses a general linear model framework that, starting with the intercept, sequentially adds drivers that most improve the fit to the response variable (i.e., number of nonnative species). This forward selection technique terminates at the model with the minimal value of Akaike's conservative information criterion (AICc) that balances goodness of fit against model complexity (Draper & Smith, 1981).

The drivers had low collinearity values, that is, Variance Inflation Factors including the number of native taxa (VIF = 1.3), NPP (1.5), mean average temperature of the site (2.1), population density within 100 km radius (2.5), and the average time it takes traffic to move 1 m (3.4). In addition, we evaluated the categorial variable NLCD land cover to account for differences among habitats. Finally, we used a second method to validate results from AICc‐based regression, using a Type III Sum of Squares General Linear Mixed Model using a Gaussian distribution (Proc GLIMMIX SAS_Institute, 2013).

## 3 RESULTS

**FIGURE 4:**
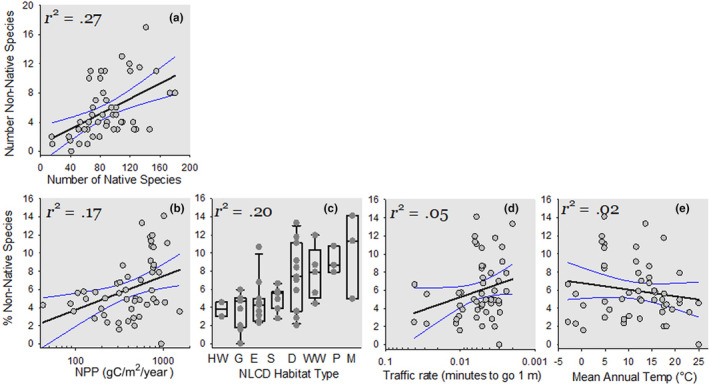
Ecological drivers of the number of nonnative invertebrates in 51 North American communities as measured from eDNA metabarcoding of NEON pitfall samples. AICc‐guided regression first identifies (a) an average of 1 nonnative for every 17 native species in a community. Subsequent plots of residuals reveal that (b) % nonnatives increase by 1% for every 180 gC/m^2^/year; (c) habitats vary threefold in their % nonnatives (G = herbaceous grasslands, HW = herbaceous wetlands, E = evergreen forest, S = Shrub/Scrublands, D = Deciduous forests, WW = woody wetlands, P = pasture/hay, M = mixed forest); (d) % nonnatives increase with the rate of traffic (note reversed *x*‐axis); and (e) % nonnatives decrease with temperature. Least squares regression (±95% CI) and partial regression coefficients provided.

**FIGURE 5:**
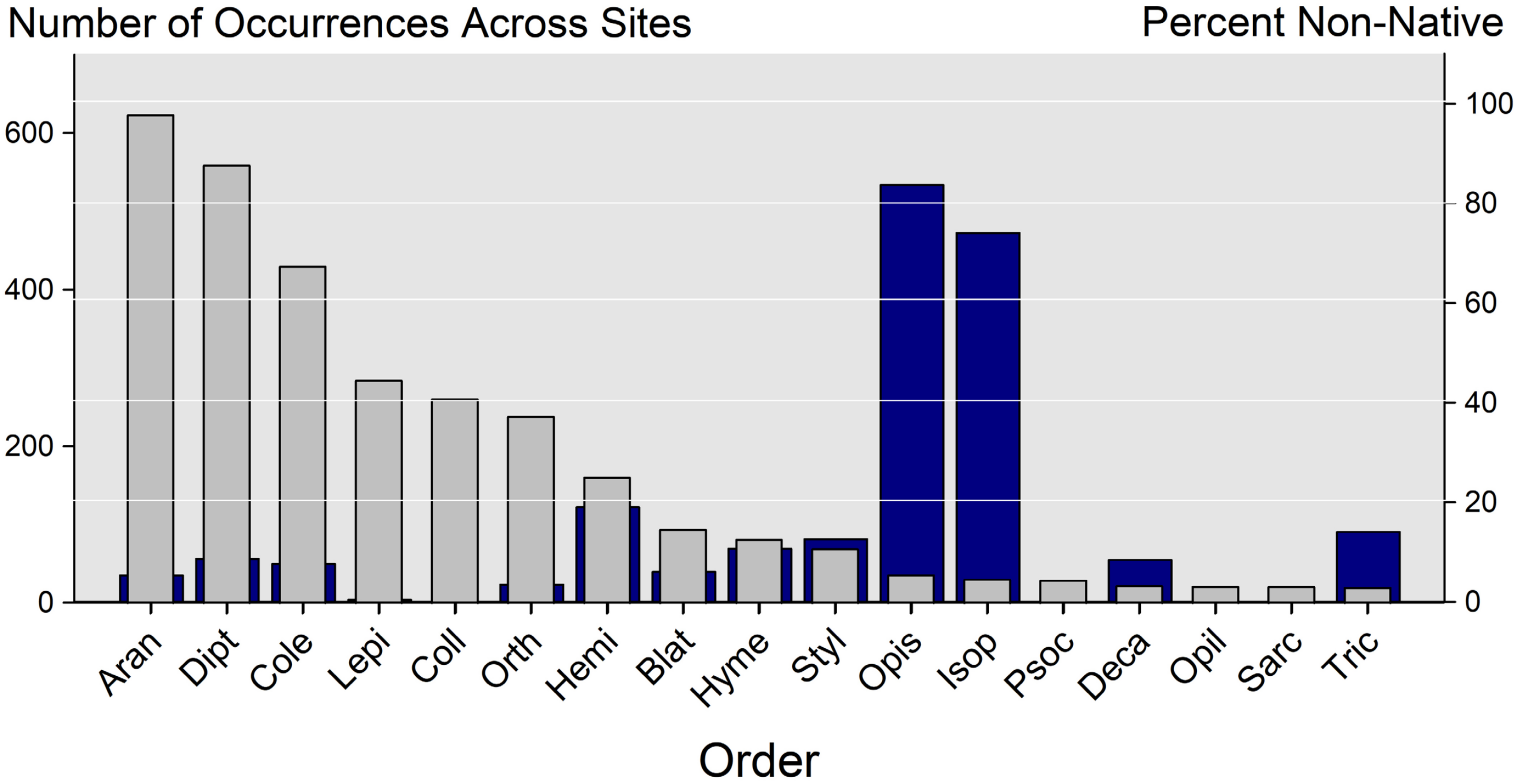
The relative frequency of orders detected via eDNA metabarcoding (gray) and the percentage that are nonnative (purple). The 17 most common taxa—each representing at least 20 species records across the 27 NEON sites—represent 93% of the individuals from a total of 69 recorded orders. Aran, Araneae; Blat, Blattoidea; Cole, Coleoptera; Coll, Collembola; Deca, Decapoda; Dipt, Diptera; Hemi, Hemiptera; Hyme, Hymenoptera; Isop, Isopoda; Lepi, Lepidoptera; Opil, Opiliones; Opis, Opisthopora; Psoc, Psocodea; Sarc, Sarcoptiformes; Styl, Stylommatophora; Tric, Trichoptera.

Metabarcoding of 51 communities yielded species richness that varied 10‐fold (mean = 88, range = 15–180) with 17 invertebrate orders representing 93% of all individuals. Nonnative species made up an average of 6% of a community (range from 0% in a Puerto Rican grassland to 14% in a Wisconsin mixed forest, Figure 4). Nonnative species were a small fraction (<5%) of the species records from the 17 orders and 51 communities (Figure 5) save for two—the earthworms and isopods—where nonnatives represented the majority of records.

Our AICc‐based test of hypotheses accounted for two‐thirds of the between‐community variation in nonnative taxonomic richness (Figure 4) and was validated by a General Linear Mixed Model (Appendix S1, Table S1). Capacity rules accounted for 34% of this variation. A community's native species richness was the strongest predictor of its nonnative richness (Figure 4), averaging one nonnative for every 14 native species (*p* < .0001, *F*_1,38_ = 41). After accounting for native diversity, a 35‐fold gradient of Net Primary Productivity accounted for another 17%. A new nonnative species was detected on average with every added 250 gC/m^2^/year of NPP (*p* = .0035, *F*_1,38_ = 9.7), posited to limit the number of individuals, a constraint to species richness a community can support.

After accounting for Capacity constraints, the AICc selection process identified two examples of Establishment constraints associated with the availability of disturbed sites and the number of propagules to colonize them. First, the eight habitat types varied three‐fold in their number of nonnatives, with herbaceous wetlands, grasslands, evergreen forest, and shrub scrub supporting ca. 5% nonnatives, while forests with deciduous trees (including mixed forests), woody wetlands, and pastures supported 7%–12% (*p* = .0009, *F*_7,38_ = 4.6). Consistent with the hypothesis that disturbed habitats are more invasible (Simberloff, 2013), pastures supported twice as many nonnatives as undisturbed grasslands. We failed to detect a posited increase in invasibility with a pitfall array's local human population density (Dawson et al., 2017); however, invasibility decreased with an indicator of the slowness of traffic flow (or “friction”, Weiss et al., 2020) at the same spatial grain (*p* = .0076, *F*_7,38_ = 7.9). Faster traffic, after accounting for native diversity, NPP, and habitat type, was associated with more nonnatives.

Finally, contrary to prediction, colder ecosystems did not restrict, but slightly enhanced, a community's number of nonnative species (Figure 4).

## 4 DISCUSSION

While the spread of nonnative species has been studied early and well on island and regional scales (Dawson et al., 2017; Sax & Gaines, 2008; Seebens et al., 2021; Simberloff, 2013) we use a network of pitfall traps and eDNA to reveal a >10‐fold variation in the number of nonnative species across 51 North American invertebrate communities. After accounting for the of role native diversity, we show evidence for the Capacity processes of Brown (1981) promoting the ability of the ecosystem to support species/populations (i.e., the drivers of native richness and NPP) and Establishment processes that describe the rate propagules are carried across the landscape (i.e., speed of traffic) and provide open space to colonize once they do (i.e., the frequency of disturbance in hay fields and pasture vs. native grassland). With some notable exceptions (earthworms and terrestrial isopods) we find that most taxa currently have relatively small (<5%) incidents of nonnative species—a pattern predicted to be temporary (Seebens et al., 2021).

The use of a continental array of traps like that of NEON for a group as large and diverse as the invertebrates was bound to also yield novel insights into the structure of invertebrate communities. First, invertebrate orders supplied widely varying fractions of records of nonnative species from 0% (e.g., Lepidoptera and Collembola) to >70% (earthworms and isopods). Some of this variation may reflect unequal representation in genomic libraries (and thus, e.g., fail to detect hidden local diversity). However, both the well‐studied butterflies and the more taxonomically challenging collembola revealed the same 0% nonnative community composition. Their phylogeny and high diversity of earthworms and isopods in the eastern North America point to a European origin (Jass & Klausmeier, 2000); their abundance suggests profound and novel rearrangements of the brown food webs in these ecosystems (Bohlen et al., 2004; Ferlian et al., 2020).

Second, some habitats—grasslands, evergreen forests, and shrub scrub—appear less invasible (medians of <6% nonnatives) than others (deciduous and mixed forests with medians 7%–11% nonnative). This suggests a role for deciduous trees in promoting invasibility. One working hypothesis is that the litter layer generated by these trees is also a major source of food for two of the most invasive taxa—earthworms and isopods. A second is that the relatively high nutrient content of hardwood leaves compared to grasses and coniferous needles (Marschner, 1995)—as well as its deposition as a uniform layer of microbe‐rich leaf litter—may, like the periodic mowing of hay fields, provide a seasonally rich resource pulse. Propagules arriving during this autumnal pulse likely find a window of reduced competition from the native community. If true, we should see similar patterns of high invasibility of deciduous forests in Europe, a source pool for North American isopod and earthworm diversity.

We also show quantitative evidence for a more direct human connection to the spread of nonnatives: hitchhiking on cars, trucks, and other vehicles. Like an earlier study on human population density as a driver of nonnative distribution of amphibians, ants, birds, freshwater fishes, mammals, vascular plants, reptiles, and spiders (Dawson et al., 2017), we reveal how variation in the isolation of our 51 communities from traffic acts as a constraint on the introduction of propagules (Von der Lippe & Kowarik, 2007). If broadly true, the reduction of traffic in national parks and reserves via gateways at entrances (Beunen et al., 2008) may be further justified as actions to slow the Homogocene.

### 4.1 Caveats

Our results come with caveats. First, eDNA is increasingly realized as a tool to quantify biodiversity (Mena et al., 2021; Taberlet et al., 2018) but not without potential noise and bias from sample capture and preservation to sequencing and bioinformatic pipelines (see review in Luo et al., 2023). As in other attempts to quantify biodiversity trends at regional to continental scales (Hallmann et al., 2017; Kaspari Yuan & Alonso, 2003; Srivastava & Lawton, 1998) standardized methodology helps. Such deviations are reduced by NEON's use of the same sampling protocols across habitats from tundra to rainforest (Levan, 2020) and our standardized bioinformatic pipelines (Rourke et al., 2022). Future developments of new primers and pipelines, growth in genomics libraries for understudied invertebrates, and comparisons of eDNA surveys with intensively studied single biotas (Mena et al., 2021) will increase the breadth, accuracy, and error estimation of this widely accessible technology.

Second one strength of our theoretical focus on Capacity and Establishment rules is their decidedly neutralist approach (Hubbell, 2001; MacArthur & Wilson, 1967) focusing on mechanisms shared among individuals regardless of identity. This is, of course, also a limitation. For example, the variation we reveal among taxa highlights the potential importance of differences in functional traits (Enders et al., 2020; Tilman, 1997). We suggest, however, that our simple neutral models are a good start which have identified the inter‐taxa variation that can highlight the existence of such functional groups and their traits.

A key challenge in comparative community ecology is the standardized sampling of the available bioclimatic and physiognomic variation at a continental scale. Toward a remedy, we used NEON's arrays of pitfall traps to characterize the richness of native and nonnative ground invertebrates across US North America. Moreover, we show the ethanol used to preserve bulk samples of insects (such as those used in documenting insect declines, Hallmann et al., 2017) to be useful in nondestructively monitoring community change. The biodiversity of local communities arises from a mixed process of adding taxa from larger source pools and delaying their extirpation (Enders et al., 2020; Kaspari Yuan & Alonso, 2003). Many of the drivers implicated here—NPP, habitat disturbance, traffic—are themselves subject to ongoing change (Wagner et al., 2021). NEON's pitfall arrays, given its proposed 30‐year lifespan, should help us evaluate projections of a large increase in arthropod nonnatives worldwide (Seebens et al., 2021) and further test, refute, and refine the list of drivers of community nonnative richness.

## AUTHOR CONTRIBUTIONS

**Michael Kaspari:** Conceptualization (equal); formal analysis (equal); writing – original draft (lead); writing – review and editing (equal). **Michael D. Weiser:** Conceptualization (equal); data curation (lead). **Cameron D. Siler:** Conceptualization (equal); methodology (equal); writing – original draft (supporting); writing – review and editing (supporting). **Katie E. Marshall:** Conceptualization (equal); writing – original draft (supporting); writing – review and editing (supporting). **Sierra N. Smith:** Investigation (equal); methodology (equal). **Katherine M. Stroh:** Investigation (equal); methodology (equal). **Kirsten M. de Beurs:** Conceptualization (equal); investigation (equal); methodology (equal); writing – review and editing (supporting).

## CONFLICT OF INTEREST STATEMENT

The authors have no competing interests.

## Supporting information


Appendix S1:

## Data Availability

The data that support the findings of this study are openly available in Open Science Framework https://osf.io/935cs/, DOI 10.17605/OSF.IO/935CS.
