# Supplementary material for: Capacity and establishment rules govern the number of nonnative species in communities of ground‐dwelling invertebrates
Source: Ecol Evol. 2024 Mar 13;14(3):e10856. doi: 10.1002/ece3.10856 (PMC10937486; doi:10.1002/ece3.10856)
Supplement: Supplementary file 1 — Appendix S1: [file ECE3-14-e10856-s001.docx]

# Appendix S1

The National Ecological Observatory Network was constructed over a five-year period (2014–2019) for a proposed 30 years of monitoring abiotic and biotic changes across the ecosystems of the United States (Alaska, Hawaii, Puerto Rico, and the 48 contiguous states). At each site, NEON identifies up to four habitat types based on NLCD land cover codes, seven of which are represented here (deciduous forest, mixed forest, evergreen forest, herbaceous grasslands, pasture/hay, shrub scrub, and herbaceous and woody wetlands , Fry et al. 2008). Ten pitfall trap arrays are distributed across the habitats and sampled across the growing season, defined as the weeks when average minimum temperatures exceed 4°C for 10 days and ending when temperatures remain below 4°C for the same period.

Each trap is a 473 mL plastic deli container *ca*. 11 cm in diameter and 7 cm deep containing 150–250 ml of 1:1 deionized water and propylene glycol (summarized in Levan 2020). Traps are placed flush in the soil with a square cover 1.5 cm above the trap. Every 14 days, traps are emptied and replaced; the contents of all traps from a given array are pooled and stored in 95% EtOH-filled 50-mL tubes (some arrays required multiple tubes).

For this analysis, we used 51 site-habitat samples from 2016. For the majority (44), each represents three pitfall samples pooled from biweekly samples taken early, in the middle, and at the end of the site’s sampling season. The remaining 7 are means of two such arrays sampled from the same site-habitat.

## Molecular methods

To identify taxa in the pitfall traps we used methods modified from current environmental DNA techniques (Taberlet et al. 2018), which allowed us to non-destructively sample DNA from the invertebrates using the ethanol in which they were preserved. All of the storage ethanol was vacuum filtered using a 50 mL Autofil® Vacuum Filtration System (Stellar Scientific) with a 0.45 µm pore size. After filtering, we used sterilized scissors to cut the filter from the tube which was then stored at -20°C.

## DNA Extraction

To extract DNA, the filters were placed on a UV-sterilized paper towel and cut into smaller pieces. These pieces were put in a sterile, 1.5 mL microcentrifuge tube and then dried in a Savant DNA 120 SpeedVac Concentrator (ThermoScientific) at low temperature for 20 minutes with lids open. After drying, we added 380 µL ATL buffer and 20 µL Proteinase K (DNeasy Kit, Qiagen Corp.) before vortexing for 20 seconds and then incubating at 55°C with low speed (500 rpm) overnight.

The next day the tubes were vortexed again for 15 seconds and the filter pieces and liquid were transferred to a Qiashredder spin column (Qiashredder Kit, Qiagen Corp.) which was spun for 5 minutes at 8000 rpm. Then, the filtrate was transferred to a sterile, 5 mL tube and 600 µL AL buffer (DNeasy Kit, Qiagen Corp.) was added. The tube was vortexed and then incubated at 70° C for 10 minutes. We then added 600 µL of chilled, 200 proof ethanol and immediately vortexed the tubes for 10 seconds. This mixture was centrifuged in a Qiamp Spin column (DNeasy Kit, Qiagen Corp.) at 8000 rpm for 1 minute. The spin column was then transferred to a clean collection tube and this process was repeated 2–3 times until all of the filtrate had passed through the spin column. The spin column was placed in a new collection tube and 500 µL AW1 buffer was added then centrifuged at 8000 rpm for 1 minute. Then, the spin column was moved to another clean collection tube and 500 µL of AW2 buffer was added and the tube spun at 15,000 rpm for three minutes. We took out the spin column and placed it in a sterile, 1.5 mL microcentrifuge tube and the DNA was eluted with 50 µL of Buffer AE (preheated to 70°C). The spin column was incubated at room temperature for 5 minutes then spun at 8000 rpm for 1 minute. After spinning the sample, we added another 50 µL of Buffer AE and incubated the tube at room temperature for 2 hours then spun for 1 minute at 8000 rpm. Then, we prepared the Inhibition Removal spin columns following the provided protocol (OneStep PCR Inhibitor Removal Kit, Zymo Research) and transferred the spin columns to a sterile, labeled microcentrifuge tube. The 100 µL of eluted DNA was added to the spin column and centrifuged at 8000 rpm for 1 minute. Finally, the spin column was removed and the lids of the microcentrifuge tubes were closed. Samples were stored in a -20° C freezer until ready for genomic library preparation.

## Metagenomic Barcoding

Following DNA extraction, we used a two-step Polymerase Chain Reaction (PCR) protocol to amplify three fragments of the mitochondrial cytochrome oxidase I gene (COI): F230R, 157, and Lep (see table S2.2, Hajibabaei et al. 2019, Rennstam Rubbmark et al. 2018) Two-step PCR eliminates separate annealing and extension steps; instead, annealing and extension are combined in a single step at a relatively low temperature (48ºC), but for a relatively long time (5 min). Initial experiments showed that two-step PCR outperformed traditional three-step PCR. PCR amplification was confirmed by gel electrophoresis and initial PCR products were cleaned with KAPA Pure Beads (Roche Sequencing Solutions). A second PCR was performed using the cleaned PCR products to attach unique Nextera paired-end indices (IDT for Illumina DNA/RNA UD Indexes, Illumina Inc.) and i5 and i7 adaptor sequences to the amplified DNA so the prepared product was able to bind to the surface of the Illumina MiSeq flow cell (Klymus et al. 2017). Next, PCR products were cleaned a second time with KAPA Pure Beads (Roche Sequencing Solutions), and then quantified using a Qubit fluorometer (Invitrogen). After quantification, all samples were normalized to 6 nM of DNA before being pooled into a sterile, 1.5 mL microcentrifuge tube. If the DNA quantity of a sample was above 6 nM, 5 mL of the sample was added to a calculated amount of sterile, laboratory grade water to dilute the sample to 6 nM. After dilution, 4 mL of the diluted PCR product was added to the final pool. If the DNA quantity of a sample was below 6 nM, no water was added to the sample and 2 mL of the PCR product was added to the final pool. Sequencing was performed at the University of Oklahoma Consolidated Core Lab on an Illumina MiSeq, producing 2 X 500 bp reads for F230R primers, 2 X 300 bp reads for 157, and 1 X 600 bp reads for Lep.

## Taxonomic Assignment

We used the command “-fastq_mergepairs” in USEARCH (Edgar 2010) to assemble paired-end reads to create consensus sequences for the two paired primers (157 and LCO). We size-filtered all primers to the expected sequence length and quality filtered (“-fastq_filter”, “-fastq_maxee”) removing any sequence with more than one error. We removed singletons (“-sortbysize”) before clustering sequences to OTUs (“-cluster_otus”) and then assigning all reads (including singletons) to OTUs (“-otutab”). We then used BLASTn (Altschul et al. 1990) to assign taxonomy to each OTU, selecting the best 10 matches between the consensus OTU sequence and NCBI databases. To set a limit for inclusion, we used linear regression of e-score on percent similarity for each primer to calculate the average e-score at 97% similarity between the OTU consensus sequence and the BLAST search. Where the best match was not determined to species in the NCBI database (e.g., order, family, genus) we used the taxonomy from matches with lower scores, provided they did not contradict any matches with better scores and met the minimum critical quality score based on 97% similarity. We refer to reads that meet these quality and taxonomic criteria as High-Quality reads (“HQreads”).

Using the results from BLASTn search, we assigned taxonomy to each consensus sequence, using Integrated Taxonomic Information System (ITIS) as a standard baseline [Retrieved between January 10, 2020 and September 30, 2020 (<http://www.itis.gov)>].

## Assigning non-native status

We queried each species name x site occurrence against online databases including Bison (BISON 2022), GBIF (Flemons et al. 2007), iNaturalist (Van Horn et al. 2018), BugGuide (Bartlett 2003), the World Spider Catalogue (Platnick 2010). Next Google Scholar searches (scholar.google.com/)began with “Genus+species+U.S. state of interest”. Lacking any hits, we proceeded to “Genus+species+United States”, then “Genus+Species+distribution”, then “Genus+species. The process was then repeated using Google (google/com). Criteria denoting non-native status varied among sources (e.g., “invasive”, “naturalized”, etc.) but only when a species occurrence at one of our pitfall array sites was identified as such, we assigned it native status.

**Table S1:** Verifying the AICc-based GLM method revealing the effects of five drivers on non-native richness using a General Linear Mixed Model (Type III Sums of Squares).

| Effect | DF | F | Pr>F |
| --- | --- | --- | --- |
| Native Species | 1 | 41.25 | <.0001 |
| Habitat type | 7 | 4.59 | 0.0009 |
| Temperature | 1 | 12.31 | 0.0012 |
| NPP | 1 | 9.71 | 0.0035 |
| Traffic Rate | 1 | 7.94 | 0.0076 |
| Error | 38 |  |  |

## **SI References**

Altschul, S. F. et al. 1990. Basic local alignment search tool. — J. Mol. Biol. 215: 403-410.

Bartlett, T. 2003. BugGuide. Net: Identification, Images, & Information For Insects, Spiders & Their Kin For the United States & Canada.

BISON 2022. Biodiversity Information Serving Our Nation (BISON). Field Museum of Natural History.

Edgar, R. C. 2010. Search and clustering orders of magnitude faster than BLAST. — Bioinformatics 26: 2460-2461.

Flemons, P. et al. 2007. A web-based GIS tool for exploring the world's biodiversity: The Global Biodiversity Information Facility Mapping and Analysis Portal Application (GBIF-MAPA). — Ecological informatics 2: 49-60.

Fry, J. et al. 2008. Completion of the National Land Cover Database (NLCD) 1992–2001 land cover change retrofit product. — US Geological Survey open-file report 1379: 18.

Hajibabaei, M. et al. 2019. COI metabarcoding primer choice affects richness and recovery of indicator taxa in freshwater systems. — PloS one 14: e0220953.

Klymus, K. E. et al. 2017. Metabarcoding of environmental DNA samples to explore the use of uranium mine containment ponds as a water source for wildlife. — Diversity 9: 54.

Levan, K. 2020. NEON user guide to ground beetle sampled from pitfall traps. NEON, Inc. Boulder, CO.

Platnick, N. I. 2010. The world spider catalog, version 10.5. — <http://research>. amnh. org/entomology/spiders/catalog/index. html

Rennstam Rubbmark, O. et al. 2018. A broadly applicable COI primer pair and an efficient single‐tube amplicon library preparation protocol for metabarcoding. — Ecology and Evolution 8: 12335-12350.

Taberlet, P. et al. 2018. Environmental DNA: For biodiversity research and monitoring. — Oxford University Press.

Van Horn, G. et al. 2018. The inaturalist species classification and detection dataset. Proceedings of the IEEE conference on computer vision and pattern recognition. pp. 8769-8778.
